# Acromegaly with initial negative oral glucose tolerance test: a case report

**DOI:** 10.1186/s13256-023-04064-z

**Published:** 2023-08-06

**Authors:** Wen-Hsuan Tsai, Ming-Nan Chien, Shuen-Han Dai, Yun-Kai Chan

**Affiliations:** 1 Division of Endocrinology and Metabolism, Department of Internal Medicine, MacKay Memorial Hospital, Taipei, Taiwan, ROC; 2 Department of Medicine, MacKay Medical College, New Taipei City, Taiwan, ROC; 3 Department of Pathology, MacKay Memorial Hospital, Taipei, Taiwan, ROC; 4 Division of Neurosurgery, Department of Surgery, MacKay Memorial Hospital, No. 92, Sec. 2, Zhongshan N. Rd., Taipei City, 10449 Taiwan, ROC

**Keywords:** Acromegaly, Pituitary macroadenoma, IGF-1, GH, Oral glucose tolerance test

## Abstract

**Background:**

Acromegaly can be diagnosed by a growth hormone value ≥ 1 µg/L following an oral glucose tolerance test. However, normal growth hormone suppression following oral glucose tolerance test may not exclude acromegaly.

**Case presentation:**

We present a case of a 55-year-old Chinese man with pituitary macroadenoma incidentally noted after a traffic accident. He reported feet enlargement in the past few years. At the beginning, elevated insulin-like growth factor-1 was noted with growth hormone value < 1 µg/L after oral glucose tolerance test. Fracture-related high insulin-like growth factor-1 was suspected. Insulin-like growth factor-1 decreased gradually but was still above the upper limit of normal . However, he suffered from dizziness 1 year later and insulin-like growth factor-1 increased again. Besides, secondary hypocortisolism developed. The size of the pituitary macroadenoma was stationary. Follow-up oral glucose tolerance test showed a growth hormone value > 1 µg/L. Endoscopic endonasal approach to the remove pituitary macroadenoma was performed subsequently. After the resection of the pituitary macroadenoma, pathology showed positive staining of growth hormone and prolactin. Insulin-like growth factor-1 normalized as well.

**Conclusions:**

Suppressed growth hormone after oral glucose tolerance test cannot exclude acromegaly, and some patients may have only mild or no clinical presentation of acromegaly. Patients with pituitary microadenoma or macroadenoma and elevated insulin-like growth factor-1 should be closely monitored for signs/symptoms of acromegaly and hypopituitarism.

## Background

Acromegaly is usually recognized with typical symptoms and signs, including characteristic coarse facial changes, hands and feet swelling, macroglossia, snoring and sleep apnea, carpal tunnel syndrome, headache, sweating, and new-onset diabetes mellitus [1]. Acromegaly can be diagnosed by measuring serum insulin-like growth factor-1 (IGF-1) and growth hormone (GH). An unsuppressed GH value ≥ 1 µg/L following an oral glucose tolerance test (OGTT) confirms the diagnosis [2]. However, discordant GH and IGF-1 values with elevated IGF-1 levels but normal GH suppression following OGTT may not exclude acromegaly [3]. Ribeiro *et al.* reported 40 patients with newly diagnosed acromegaly, with 33% of which having a GH nadir < 1 µg/L, and 18% of which having a GH nadir < 0.4 µg/L [4]. On the other hand, a GH nadir between 0.4 and 1 µg/L during OGTT does not certainly indicate a pituitary microadenoma [5]. The interpretation of IGF-1 and GH after OGTT should be done carefully according to clinical presentation and relevant history. Herein, we present the case of a 56-year-old man with acromegaly, who had persistent elevated IGF-1 and initial suppressed GH value < 1 µg/L after OGTT. We aimed to reinforce the importance that a suppressed GH value may not totally exclude acromegaly.

## Case presentation

**Fig. 1:**
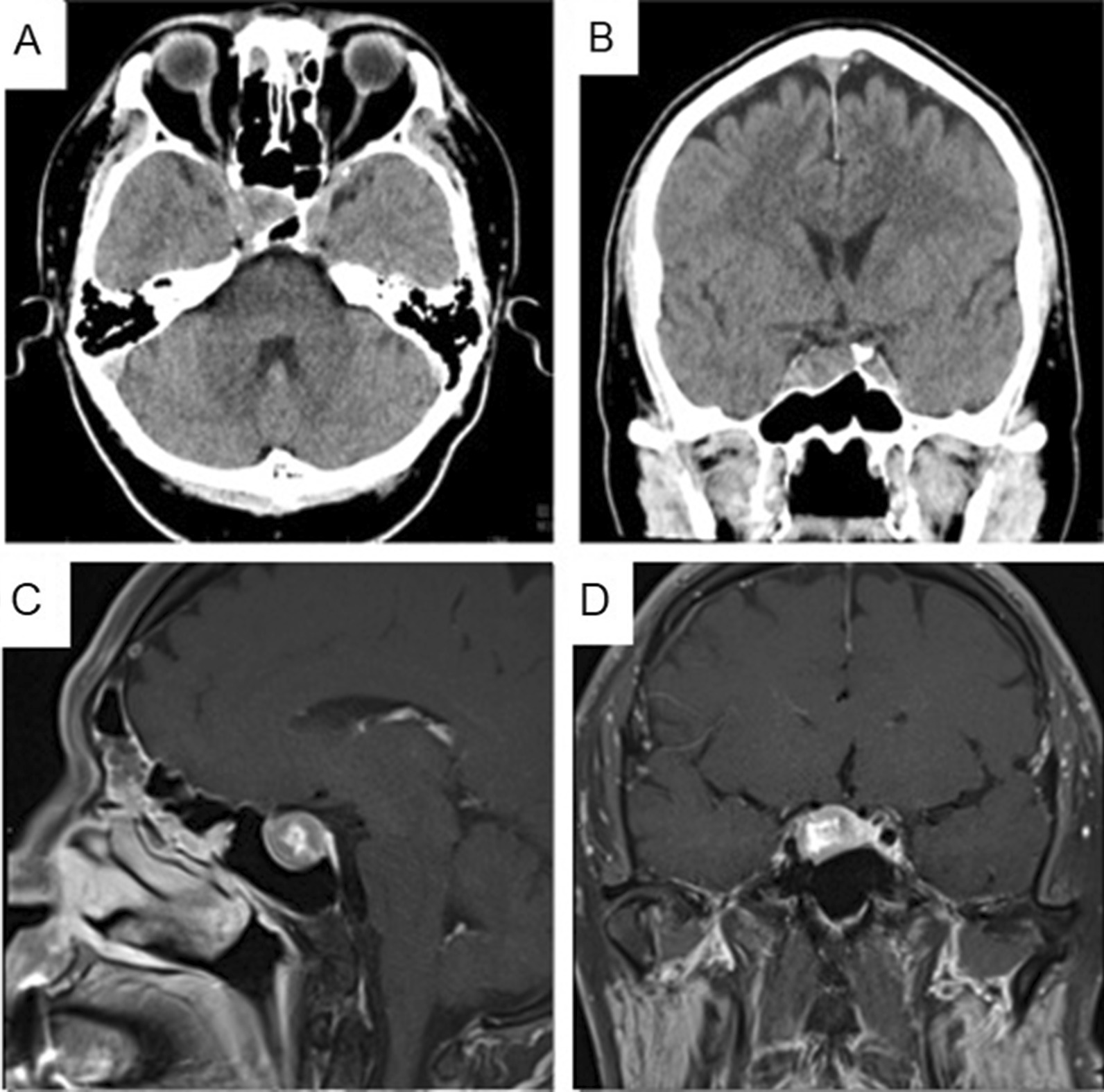
Image of pituitary macroadenoma. **A**, **B** Computed tomography (CT) and **C**, **D** Magnetic resonance imaging (MRI) revealed a 1.8 cm × 1.6 cm × 1.6 cm heterogeneously enhancing lesions in right pituitary gland, resulting in left shift of the pituitary stalk. Invasion of medial portion of right cavernous sinus was likely

**Fig. 2:**
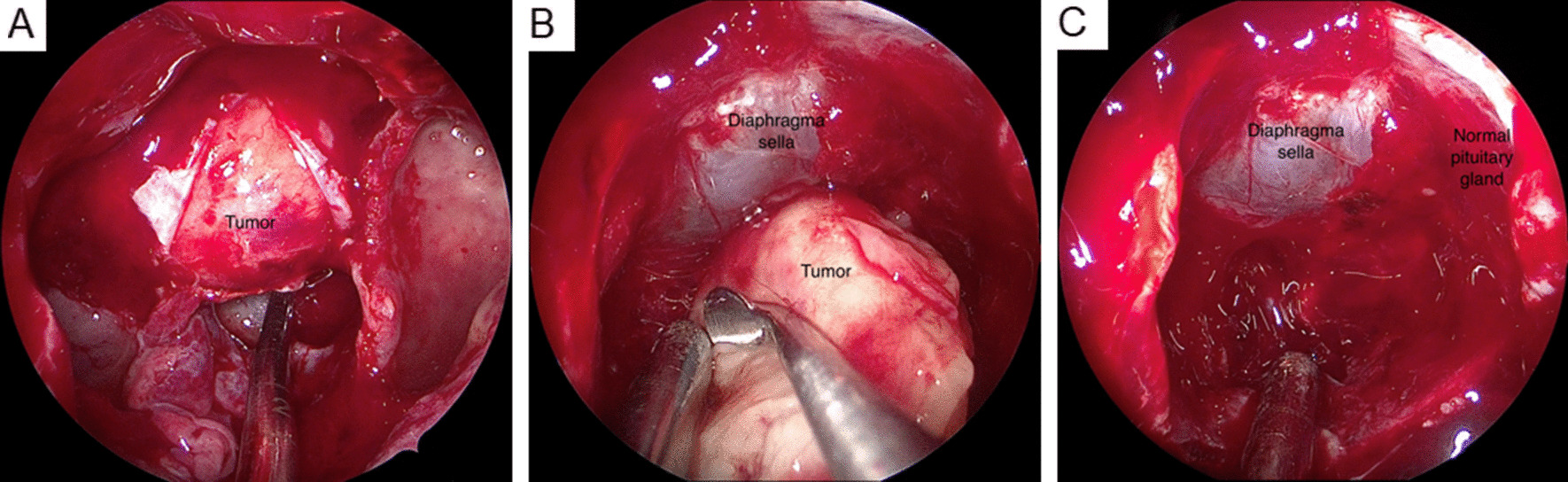
Endoscopic endonasal approach to remove pituitary macroadenoma. **A** The dura was incised, and the tumor was exposed. The tumor was yellow and soft. **B** Extracapsular dissection between tumor pseudocapsule and diaphragma sella was performed. **C** The normal pituitary gland was identified and kept intact without injury

**Fig. 3:**
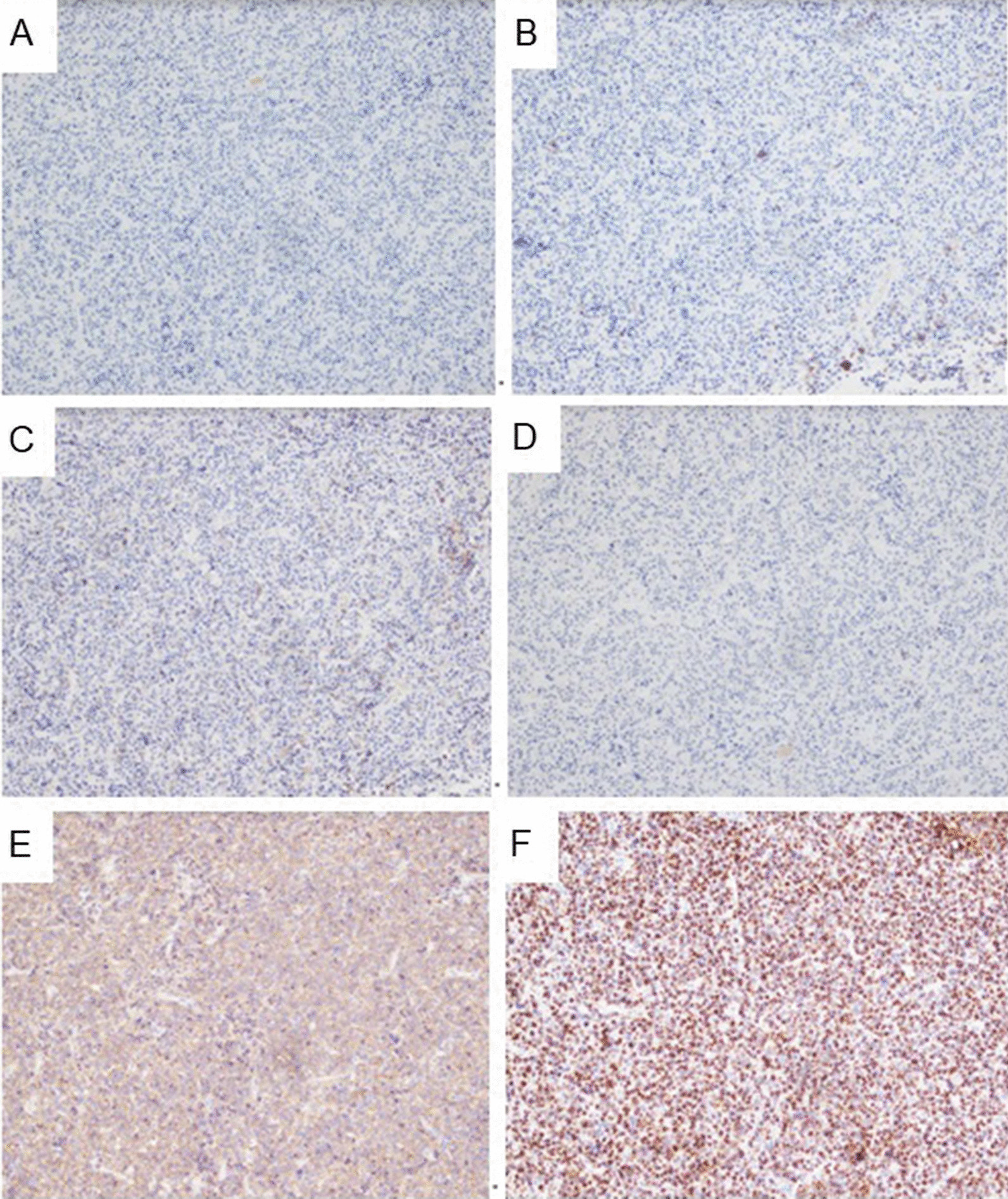
Immunohistochemical staining for pituitary macroadenoma. **A** Adrenocorticotropic hormone (ACTH): negative **B** Prolactin: focal positive **C** GH: focal positive **D** Ki-67: positive **E** synaptophysin: positive **F** CAM 5.2: positive

This patient was a 55-year-old Chinese man who had a history of chronic hepatitis B and prediabetes. He had no family history of hypertension nor diabetes. He worked in a supermarket and had to carry heavy loads sometimes. He had been a smoker for over 40 years and seldom consumed alcohol. No medication was received previously. He suffered from a traffic accident with multiple ribs fracture and scapula fracture in September 2021. The initial blood pressure was 108/64 mmHg, and body temperature was 36 °C, while heart rate was 82 beats per minute. The patient was alert upon admission, with Glasgow Coma Scale (GCS) of E4M6V5. Physical examination showed right shoulder and chest wound, with right arm and chest tenderness. Right shoulder range of motion was limited, while the sensation of right arm was intact. Sensation over other extremities was normal. There was no visual field defect, with normal pupil size, light reflex, eye movement, muscle strength, and muscle tone. He had normal gait and the deep tendon reflex was intact. Pituitary macroadenoma was incidentally noted on brain computed tomography (CT) (Fig. 1). Sella magnetic resonance imaging (MRI) revealed a 1.8 cm × 1.6 cm × 1.6 cm heterogeneously enhancing lesions in the right pituitary gland, resulting in a left shift of the pituitary stalk. Invasion of the medial portion of the right cavernous sinus was likely (Fig. 1). No visual impairment was noted after the examination. The endocrine profile was presented in Table 1. Prolactin level was not diluted during the treatment course. Since elevated IGF-1 was noted, OGTT was performed, and GH was suppressed (0.45 µg/L). He mentioned that feet enlargement had been noted in the past few years. However, no facial difference was noted when compared with a previous photo. There was no frontal bossing, macroglossia, nor prognathism. Hence, elevated IGF-1 was initially suspected related to bone healing. Follow-up IGF-1 level decreased gradually but was still above the upper limit of normal (ULN) (Table 1). One year later, he complained of dizziness, and laboratory test showed secondary hypocortisolism and elevated IGF-1 (Table 1). The size of the pituitary macroadenoma was stationary. Repeat OGTT test showed a GH value of 1.44 µg/L (Table 1). Stress dose of hydrocortisone 100 mg every 8 hours was given intravenously and an endoscopic endonasal approach to remove pituitary macroadenoma was performed smoothly (Fig. 2). The tumor was soft, and extra-capsular dissection was used to remove the tumor completely. The normal pituitary gland was identified during the operation and kept intact without injury. The pathology report demonstrated focal positive levels of GH and prolactin (Fig. 3). Acromegaly was diagnosed. Hydrocortisone 100 mg every 8 hours was given for 3 days, and then tapered to 100 mg every 12 hours for 2 days. Hydrocortisone was then shifted to cortisone 25 mg twice per day. IGF-1 level normalized 3 weeks after the operation. He visited our clinic every 3 months and there was no dizziness nor headache. Cortisone dosage was tapered down gradually and then discontinued since there was no symptom or sign of adrenal insufficiency and the cortisol level was normal. The IGF-1 level was 186 ng/mL and the GH level was < 0.03 µg/L 6 months after the operation. No recurrence of acromegaly was noted.

**Table 1:** Laboratory test for pituitary macroadenoma

Baseline variable (September 2021)	Data	Reference range
Hemoglobin (g/dL)	14.6	13–18
Platelet (10^3^/μL)	165	140–450
WBC (10^3^/μL)	5.5	4.0–10.0
Glucose (mg/dL)	97	70–99
Total bilirubin (mg/dL)	0.4	0.3–1.2
Aspartate aminotransferase (IU/L)	36	15–41
Blood urea nitrogen (mg/dL)	19.4	8–20
Creatinine (mg/dL)	1.1	0.4–1.2
Sodium (mEq/L)	141.7	136–144
Potassium (mEq/L)	4.0	3.5–5.1
Cortisol (μg/dL)	11.86	9.52–26.21
ACTH (pg/mL)	31.26	10.00–70.00
FREE-T4 (ng/dL)	1.18	0.89–1.79
TSH (μIU/mL)	1.98	0.25–4.00
Prolactin (ng/mL)	7.69	1–18
LH (mIU/mL)	2.74	1.8–8.4
FSH (mIU/mL)	2.10	2.2–10
Testosterone (ng/mL)	1.90	2.51–10.63
GH (ng/mL)	2.29	< 5.0
IGF-I (ng/mL)	408.52	94–262
OGTT test		
GH nadir (ng/mL)	0.45	< 1
1 mg overnight dexamethasone suppression test		
Cortisol (μg/dL)	1.18	< 1.8
Follow-up		
IGF-I (ng/mL) (November 2021)	359.52	94–262
IGF-I (ng/mL) (March 2022)	342.48	94–262
IGF-I (ng/mL) (September 2022)	411.76	94–262
OGTT test (October 2022)		
GH nadir (ng/mL)	1.44	< 1
Cortisol (μg/dL)	6.76	9.52–26.21
ACTH (pg/mL)	6.98	10.00–70.00
Postoperation		
IGF-I (ng/mL) (November 2022)	171.53	94–262

ACTH, Adrenocorticotropic hormone; TSH, Thyroid-stimulating hormone; LH, Luteining hormone; FSH, Follicle-stimulating hormone; GH, Growth hormone; IGF-1, Insulin-like growth factor-1; OGTT, Oral Glucose Tolerance Test

## Discussion

We present a case of a patient, who suffered from a traffic accident, and a pituitary macroadenoma was incidentally noted by imaging. He also reported foot enlargement in the past few years. In the beginning, elevated IGF-1 was noted with a GH value < 1 µg/L after OGTT. Fracture-related high IGF-1 was suspected. IGF-1 decreased gradually but was still above the ULN. However, he suffered from dizziness one year later, and IGF-1 increased. Additionally, secondary hypocortisolism developed. Follow-up OGTT showed a GH value > 1 µg/L. After resection of the pituitary macroadenoma, pathology showed positive staining of GH and prolactin. The present case demonstrated that a GH nadir < 1 µg/L may not exclude the diagnosis of acromegaly, and the GH nadir may be different at different measurement.

In general, biochemical diagnosis of acromegaly is based on the elevation of IGF-1 concentrations and the absence of GH suppression after OGTT [6]. Previous study revealed that GH nadir values are assay specific and should be adjusted by age, sex, and body mass index (BMI) [7]. IGF-1 concentrations are usually slightly lower in women than in men [8]. Inadequately established reference ranges and assay interference may lead to falsely elevated IGF-1 concentrations [3]. The OGTT cutoff has been revised to 0.4 ng/mL, depending on the assay used, since more modern assays can now detect lower GH concentrations [9]. On the other hand, about 25%–30% of patients demonstrate a paradoxical increase in GH after OGTT, which is potentially related to the release of glucose-dependent insulinotropic polypeptide (GIP) from duodenal K cells signalling through its receptors on the GH-secreting adenoma [10].

Subclinical acromegaly was proposed by Shimon *et al.* [1]. The definition included baseline IGF-1 1.3-fold greater than the ULN and twofold less than the ULN, one sign or symptom of acromegaly (headache, sweating, joint symptoms, swelling), up to two associated comorbidities of acromegaly (diabetes, hypertension, sleep apnea, heart disease, hypopituitarism), and a pituitary adenoma on imaging [1]. Based on the criteria above, our patient could be diagnosed with subclinical acromegaly, with initial IGF-1 level being 1.6-fold greater than the ULN, having enlargement of the feet, prediabetes, and a pituitary adenoma. A study of 24 patients who received an operation with a diagnosis of somatotroph adenomas showed that 45.8% of the patients had a classic presentation of acromegaly, while 16.7%, 33.3%, and 4.2% of patients had subtle, clinically silent symptoms and silent somatotroph adenomas [11]. The immunohistochemical staining for GH of adenomas from eight patients with clinically silent somatotroph adenomas showed strong staining, some diffuse, and others scattered [11]. Our patient had subtle presentation of acromegaly and the pathology revealed focal positive levels of GH.

Nonfunctioning macroadenomas account for about 80% of all pituitary macroadenomas [12]. In our patient, since GH was suppressed by OGTT, the elevated IGF-1 level was suspected to be related to the fracture event. Serum IGF-1 correlates to the quality of fracture healing, and impairment of the somatotropic axis might be involved in the pathophysiological mechanism determining delayed or failed fracture healing [13]. IGF-1 revealed no significant changes over 6 months after the operation for fracture [13]. When the elevated IGF-1 related to fracture healing is expected to normalize after occurrence of a fracture still warrants further research.

A longer interval between acromegaly onset and diagnosis has been associated with higher all-cause mortality and a higher number of comorbidities, reinforcing the importance of early detection and prompt management [14]. Surgical resection of the adenoma is the first-line approach for most patients, with normalization of age-adjusted IGF-1 concentrations and reduction of GH concentrations to < 1 µg/L (or < 0.4 µg/L with ultrasensitive assays) being the primary therapeutic target [2]. Medical treatment is indicated mostly for persistent disease after surgery or for those patients who are unlikely to receive surgery, while radiotherapy is typically reserved as a third-line option in patients who have no adequate response to medical therapy, or have a large, invasive tumor remnant [2].

## Conclusion

Suppressed GH after OGTT cannot exclude acromegaly, and some patients may have only mild or no clinical presentation of acromegaly. Patients with pituitary microadenomas or macroadenomas and elevated IGF-1 should be closely monitored for symptoms/signs of acromegaly and hypopituitarism.

## Data Availability

The datasets used and/or analyzed during the current study are available from the corresponding author on reasonable request.

## Abbreviations

IGF-1: Insulin-like growth factor-1
GH: Growth hormone
OGTT: Oral glucose tolerance test
CT: Computed tomography
MRI: Magnetic resonance imaging
ULN: Upper limit of normal
